# In-depth circulating tumor DNA sequencing for prognostication and monitoring in natural killer/T-cell lymphomas

**DOI:** 10.3389/fonc.2023.1109715

**Published:** 2023-02-10

**Authors:** Jin Ju Kim, Hyun-Young Kim, Zisun Choi, So yoon Hwang, Hansol Jeong, Jong Rak Choi, Sang Eun Yoon, Won Seog Kim, Sun-Hee Kim, Hee-Jin Kim, Sang-Yong Shin, Seung-Tae Lee, Seok Jin Kim

**Affiliations:** ^1^ Department of Laboratory Medicine, Yonsei University College of Medicine, Seoul, Republic of Korea; ^2^ Department of Laboratory Medicine and Genetics, Samsung Medical Center, Sungkyunkwan University School of Medicine, Seoul, Republic of Korea; ^3^ Dxome Co. Ltd, 8, Seongnam-si, Gyeonggi-do, Republic of Korea; ^4^ Division of Haematology and Oncology, Department of Medicine, Samsung Medical Center, Sungkyunkwan University School of Medicine, Seoul, Republic of Korea; ^5^ Department of Health Sciences and Technology, Samsung Advanced Institute for Health Sciences and Technology, Sungkyunkwan University, Seoul, Republic of Korea; ^6^ Department of Laboratory Medicine, Korea Worker’s​ Compensation & Welfare Service, Taebaek Hospital, Taebaek-si, Gangwon-do, Republic of Korea

**Keywords:** liquid biopsy, Extranodal natural killer/T cell lymphoma, biomarker, CtDNA, molecular biomarker

## Abstract

**Background:**

Epstein–Barr virus (EBV) quantitation and current imaging modalities are used for diagnosis and disease monitoring in Extranodal NK/T cell lymphoma (ENKTL) but have limitations. Thus, we explored the utility of circulating tumor DNA (ctDNA) as a diagnostic biomarker.

**Methods:**

Through in-depth sequencing of 118 blood samples collected longitudinally at different time points from 45 patients, we examined the mutational profile of each sample, estimated its impact on the clinical outcome, and assessed its role as a biomarker in comparison with EBV DNA quantitation.

**Results:**

The ctDNA concentration was correlated with treatment response, stage, and EBV DNA quantitation. The detection rate of ctDNA mutation was 54.5%, with *BCOR* (21%) being the most commonly mutated gene in newly diagnosed patients; *TP53* mutation (33%) was the most prevalent in patients that experienced a relapse. Additionally, patients in complete remission exhibited a rapid clearance of ENKTL-related somatic mutations, while relapsed patients frequently presented with persisting or emerging mutations. We detected ctDNA mutations in EBV-negative patients (50%) and mutation clearance in EBV-positive patients in remission, suggesting ctDNA genotyping as an efficient complementary monitoring method for ENKTL. Additionally, mutated *DDX3X* (PFS HR, 8.26) in initial samples predicted poor outcome.

**Conclusion:**

Our results suggest that ctDNA analysis can be used to genotype at diagnosis and estimate the tumor burden in patients with ENKTL. Furthermore, ctDNA dynamics indicate the potential use of testing it to monitor therapeutic responses and develop new biomarkers for precision ENKTL therapy.

## Background

Extranodal natural killer/T-cell lymphoma (ENKTL) is an aggressive subtype of non-Hodgkin lymphoma and the Epstein–Barr virus (EBV), which is believed to play an important role in lymphomagenesis (1), is usually detected in malignant cells. Despite an improvement in treatment strategies including non-anthracycline-based chemotherapy, the disease recurs or progresses in more than 50% of patients with ENKTL (2, 3).

Considering the high probability of relapse even after complete resolution of tumors, the detection of minimal residual disease using imaging studies is fundamental for predicting relapse and developing risk-adapted treatment strategies. However, current imaging diagnostic modalities have low sensitivity and specificity, making it difficult to detect early recurrence or progression and identify dynamic tumor responses and time-wise evolution of clones (4). Blood EBV-DNA copy numbers are widely used as an indirect biomarker in ENKTL because they can be correlated with tumor burden. However, not all patients are EBV-positive, and EBV DNA can be released from necrotic tumor cells rather than living cells (5, 6). Therefore, a new diagnostic method is needed to overcome the limitations of current monitoring modalities.

Several studies have reported various somatic gene mutations associated with ENKTL (7–9); however, a molecular classification based on genetic alterations has not yet been established for these tumor cells. A recent multi-omics study suggested new molecular ENKTL subtypes according to mutation profiles including *TP53* and *MGA* mutations and showed differing outcomes and survival rates among these subtypes (10). Thus, genetic evaluation of tumor biopsy samples prior to treatment may help predict the prognosis of ENKTL. However, a biopsy is not always possible, and the predictive value of archived tissue samples may be influenced by intra-patient heterogeneity, mainly according to the site and timing for tumor tissue sample collection.

Liquid biopsy using circulating tumor DNA (ctDNA) in plasma may be a proxy for the biological nature of tumor tissues. Recent advances in NGS technology can be applied to analyze ctDNA and facilitate cancer diagnosis, surveillance, and prognosis (11). Thus, ctDNA has been suggested as a biomarker, with several studies showing significant associations between ctDNA and relapse in diffuse large B-cell lymphoma (DLBCL), mantle cell lymphoma, and Hodgkin’s lymphoma (12–14).

In this study, we aimed to explore the clinical relevance of ctDNA at the time of diagnosis, relapse, and ENKTL progression using archived plasma samples from a prospective cohort study. The concordance of plasma ctDNA mutations with those of tumor biopsies was analyzed using 171 genes selected according to therapeutic, prognostic, and diagnostic properties (7, 15–18) (Additional file: **Table S1**). In addition, the effectiveness of ctDNA as a biomarker was compared with that of EBV DNA in patients with ENKTL using longitudinal blood samples serially collected during follow-up.

## Materials and methods

### Patients and study design

**Figure 1:**
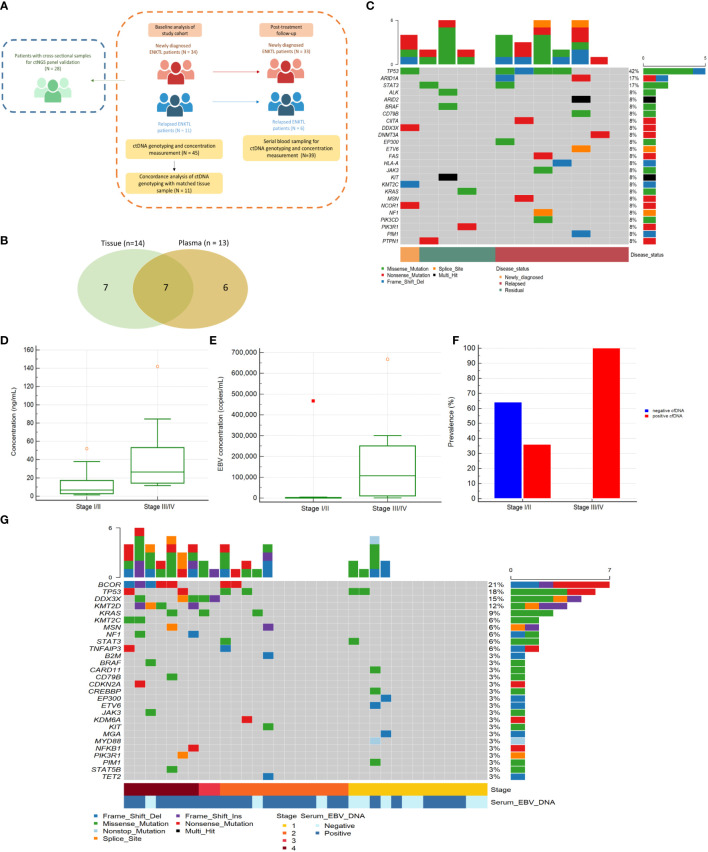
**(A)** Schematic diagram of patient enrollment and specimen collection **(B)** Concordant and discordant alterations detected in plasma and tissues. Venn diagram representing the number of mutations detected in tissue biopsy samples only, in plasma samples only (ctDNA), and in both samples. **(C)** Mutational profile of 12 ENKTL patients samples for ctDNA NGS panel validation. Heatmap showing individual non-synonymous Tier I/II somatic mutations detected in the ctDNA samples for panel validation. The top horizontal bar graph shows the gene mutation frequency. **(D)** Comparison of baseline ctDNA concentration between disease stages among 34 patients initially diagnosed with ENKTL. Comparison of median ctDNA concentrations between early-stage patients (stage I/II, n = 25) and advanced-stage patients (stage III/IV, n = 9). **(E)** Comparison of median EBV quantitation between early-stage patients (n = 25) and advanced-stage patients (n = 9). **(F)** Comparison of ctDNA detection between the advanced-stage and early-stage groups in 34 newly diagnosed ENKTL patients. Of the 34 patients, ctDNA was detected in 100.0% (n = 9) of the samples from patients with advanced-stage disease but only in 36.0% (n = 9) of samples from patients with early-stage disease. **(G)** Non-synonymous Tier I/II somatic variant detection in 34 newly diagnosed ENKTL patients from the study cohort.

This study included patients with ENKTL who were prospectively enrolled at Samsung Medical Center (Seoul, South Korea) between March 2017 and December 2020 (prospective cohort study for lymphoma: NCT03117036). The inclusion criteria were radiological and pathological diagnoses of ENKTL according to the WHO classification of tumors with hematopoietic and lymphoid tissue criteria (19): 1) aged ≥18 years at the time of diagnosis; relapsed or newly diagnosed with primary tumor invasion and/or regional lymph node involvement, 2) willing to offer peripheral blood samples prior to treatment, during treatment, and during surveillance, and 3) agreement to provide written informed consent to participate in this study. A total of 73 patients fulfilled the inclusion criteria and were enrolled in the study. First, we evaluated the ctDNA analysis platform with 34 cross-sectional archived samples obtained from 28 patients at various disease states including remission (n = 22), relapse or progression (n = 7), partial response to therapy (n = 4), and at diagnosis (n = 1). Second, the utility of ctDNA testing was evaluated in the study cohort, consisting of 34 newly diagnosed and 11 relapsed patients. Serial blood samples collected from baseline (pre-treatment or patients in relapse prior to the state of second-line chemotherapy) to the time of completion of planned treatment, disease relapse, or progression were used to compare the efficacy of ctDNA testing to that of EBV DNA for prediction of treatment outcome (**Figure 1A**).

### Clinicopathological features

Clinicopathological data of the patients were retrieved from electronic medical records and comprised age, sex, date of diagnosis, date of follow-up, prognostic index for natural killer cell lymphoma-EBV (PINK-E), International Prognostic Index score, Eastern Cooperative Oncology Group performance score, pathological information on ENKTL tissue, available NGS tissue biopsy results, and relevant laboratory findings including whole blood EBV quantitation and lactate dehydrogenase level. The Ann Arbor staging system was used for stage assessment. Imaging studies including CT, PET/CT, and magnetic resonance imaging were used to assess stage, extranodal involvement, and recurrence. The survival status of all enrolled patients was updated regularly, and the last update regarding survival and disease status was completed in January 2022.

### Sequencing and data analysis

DNA was extracted from plasma and matched FFPE tissue samples. DNA from peripheral blood mononuclear cells (PBMC) was extracted using the QIAamp DNA Mini Kit (QIAGEN, Hilden, Germany), quantified using the TapeStation 4150 (Agilent Technologies, Santa Clara, CA, USA), and fragmented to a mean size of 250 base pairs using a sonicator (Diagnode, Lausanne, Switzerland). DNA in plasma was extracted using Magnetic Circulating DNA Maxi Reagent (Dxome, Sungnam, South Korea).

The size of ctDNA was measured using the TapeStation 4150. The concentration of isolated DNA was measured using a Qubit 4.0 Fluorometer (Thermo Fisher Scientific, Waltham, MA, USA). A total of 30 ng of plasma DNA was used for library preparation; in samples with low DNA quantities, the entire DNA extract was used. Library preparation was performed using the DxSeq Library prep reagent (Dxome, Sungnam, South Korea) according to the manufacturer’s instructions. Targeting an average sequencing depth of 35,000× per sample, sequencing was done on the NovaSeq 6000 platform (Illumina, San Diego, CA, USA). For each sample, PBMCs were sequenced as germline-matched controls using identical panel and library kits, targeting an average depth >2,500×.

### Genotyping of ctDNA interpretation

We used the PiSeq and DxSeq softwares (Dxome) for variant identification and annotation, respectively, and additionally searched mutation databases and literature. Variants detected in both ctDNA and PBMCs were considered germline variants or variants of clonal hematopoiesis of indeterminate potential (CHIP). All somatic variants were categorized into Tier I/II, Tier III, or Tier IV according to the recommendation from AMP/ASCO/CAP guideline (20). All variants were visually inspected using the Integrative Genome Viewer (Broad Institute of MIT and Harvard, Cambridge, MA, USA) to exclude false positives. Owing to the lower sequencing coverage of PBMC (2,500×) compared to that of ctDNA (35,000×), potential CHIP variants may be missed in PBMC samples. Therefore, ctDNA-only variants identified in *DNMT3A*, *TET2*, and *ASXL1* with allele frequencies below 1% were also considered to be CHIP. The variant allele frequency (VAF; %) was calculated as the percentage of sequencing reads of a specific DNA variant divided by the overall coverage at that locus.

### Statistical analysis

Statistical analyses were performed using MedCalc version 18.2.1 (MedCalc Software; Mariakerke, Belgium). For continuous data, the Shapiro–Wilk test was used to detect departures from normality; variables were compared using the Mann–Whitney *U* test. The correlation between ctDNA concentration and EBV quantitation was determined using Spearman’s correlation analysis. The chi-square test or Fisher’s exact test was used to assess differences in the detection of ctDNA between groups. Progression-free survival (PFS) was measured from baseline (before the start of chemotherapy) to the date of progression, death, or the last follow-up. Overall survival (OS) was measured from the date of the initial sample draw prior to the start of chemotherapy to the date of death or the last follow-up. Survival analysis was performed using the Kaplan–Meier method. Statistical significance was defined as *P* < 0.05.

## Results

### ctDNA NGS panel evaluation for gene alterations detection

The concordance between tissue-based and ctDNA-based genotyping was analysed in 11 cases. Seven Tier I/II mutations were observed in both plasma and paired tissue, while another seven mutations were found only in tissue and six were found only in plasma (**Figure 1B** and Additional file: **Table S2**).

A total of 34 blood samples (average sequencing depth: 21,937.7×, Additional file: **Table S3**) from 28 patients at different time points were also examined. Most samples (33/34, 97.1%) were obtained during treatment or relapse. Oncogenic or druggable Tier I/II mutations were detected in 10 of 12 samples (83.3%) in patients with persistent disease or disease progression, while such mutations were not found in 22 samples from patients who achieved complete response. *TP53* was the most frequently mutated gene (5/12, 42.0%) (**Figure 1C**). Altogether, these data indicate that our ctDNA analysis platform may be a useful tool for ENKTL diagnosis and monitoring.

### Clinical characteristics of the study cohort

In the study cohort, 45 patients were enrolled. The median age at the time of enrolment was 55 years (range, 22–80 years); 64.4% (29/45) of patients were male (**Table 1**). The majority of patients was in stage I/II (71.1%), and 31 (68.9%) among them had a detectable level of EBV DNA in blood (**Table 1**). According to the PINK-E risk stratification, 21 (46.7%) patients belonged to intermediate- or high-risk groups (**Table 1**). Newly diagnosed patients with ENKTL at stage I/II involving the nasal area received concurrent chemo radiotherapy followed by two cycles of VIDL (etoposide, ifosfamide, dexamethasone, and L-asparaginase) chemotherapy. Advanced-stage patients received SMILE (steroid, methotrexate, ifosfamide, L-asparaginase, and etoposide) chemotherapy, whereas gemcitabine-containing chemotherapy or immune checkpoint inhibitors were used for relapsed or refractory disease.

**Table 1:** Characteristics of 45 ENKTL patients in the study.

Characteristic		Patients, n (%)
**Sex**	Male	29 (64.4)
	Female	16 (35.6)
**Age (year, median age: 55, range: 22 - 80)**	≤ 60	31 (68.9)
	> 60	14 (31.1)
**Disease status**	Relapsed	11 (24.4)
	Newly diagnosed	34 (75.6)
**ECOG PS**	0-1	43(95.6)
	2	2 (4.4)
**Ann Arbor Stage**	I-II	32 (71.1)
	III-IV	13 (28.9)
**Primary tumor localization**	Nasal	30 (66.7)
	Non-nasal	15 (33.3)
**Bone marrow involvement**	Presence	6 (15.6)
	Absence	39 (84.4)
**B symptoms**	Presence	7 (15.6)
	Absence	38 (84.4)
**Serum LDH**	Elevated	26 (57.8)
	Not elevated	19 (42.2)
**Blood EBV DNA**	Detected	31 (68.9)
	Not detected	14 (31.1)
**Serum β2 microglobulin**	< 2·8 mg/L	25 (55.6)
	≥ 2·8 mg/L	20 (44.4)
**IPI risk**	Low/Low-Intermediate	25 (55.6)/10 (22.2)
	High-Intermediate/High	7 (15.6)/3 (6.7)
**PINK-E risk**	Low	24 (53.3)
	Intermediate/High	21 (46.7)

EBV, Epstein-Barr virus; ECOG PS, Eastern Cooperative Oncology Group performance status score; IPI, International Prognostic Index; LDH, lactate dehydrogenase; PINK-E, prognostic index for natural killer cell lymphoma–Epstein-Barr virus.

### ctDNA concentrations as a tumor burden indicators in ENKTL

A total of 118 plasma samples including 45 at baseline, 56 during treatment and surveillance, and 17 at the time of progression were collected (average sequencing depth: 25,076.9×, Additional file: **Table S3**). The concentrations of total DNA isolated from plasma were highly variable (range, 0.39–511.30 ng/mL), and the median concentration of plasma DNA in pre-treatment or relapsed/refractory samples was 10.66 ng/mL, which was significantly higher than that in complete remission samples (4.71 ng/mL; Mann–Whitney test, *P* = 0.001). Compared to patients in early-stage disease (stage I/II), those in advanced-stage disease (stage III/IV) had higher plasma DNA concentration (26.55 vs. 7.97 ng/mL; *P* = 0.003) and blood EBV DNA titer (115,224 vs. 442 copies/mL; *P* = 0.002) at initial diagnosis prior to treatment (**Figures 1D, E**). Accordingly, the EBV DNA titer in blood was significantly associated with ctDNA concentration (*R^2 =^* 0.558; *P <*0.0001).

### Genotyping of ctDNA at diagnosis

Baseline ctDNA testing of initial samples from 34 newly diagnosed showed a detection rate of 54.5%. ctDNA mutations were more frequently observed in patients with advanced-stage disease than in those with early-stage disease (**Figure 1F**); the presence of a mutation did not correlate with detection of EBV DNA in blood (*P*=0.827). *BCOR* (21%) was the most commonly mutated gene, followed by *TP53* (18%), *DDX3X* (15%), and *KMT2D* (12%) (**Figure 1G**). The median number of mutations per sample was 1.0 (range; 0–6). The median VAF of ctDNA mutations was 2.7% (interquartile range [IQR]; 0.3%–21.4%), and the majority (79.2%) had allele frequencies exceeding 1.0%.

From a matched analysis using PBMCs as germline-matched controls, 16 among the 34 newly diagnosed patients harbored 21 variants suspicious for CHIP (Additional file: **Table S4**). *DNMT3A* (61.9%, 13/21) was the most frequently mutated gene, followed by *KMT2C* (14.3%, 3/21). The median VAF of CHIP variants was 0.9%, ranging from 0.1% to 7.2%. Nine (56.3%) among the 16 patients with CHIP variants, harbored Tier I/II mutations other than CHIP. The remaining seven patients with only CHIP variants showed no disease progression.

### Comparison of ctDNA mutations between newly diagnosed and relapsed patients

**Figure 2:**
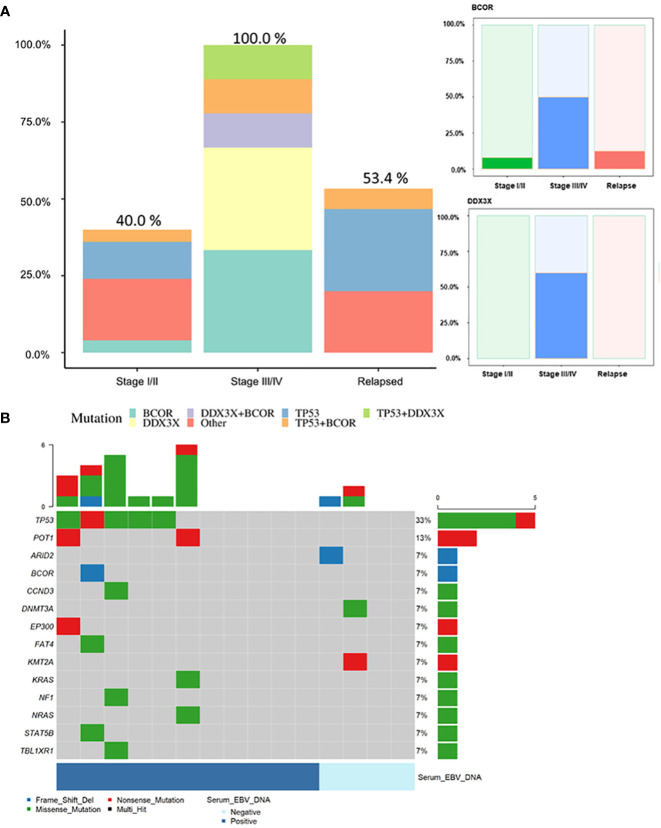
**(A)** Frequencies of genetic mutations in each of the *TP53, DDX3X*, and *BCOR* genes and other genes according to patient clinical status. Mutations of *DDX3X* and *BCOR* showed higher frequency in advanced-stage compared to early-stage or relapsed patients (*P* = 0.01 and *P* < 0.001, respectively). **(B)** Mutational profile of samples from patients with relapsed ENKTL (n = 15). Number and prevalence of mutated cases. Samples included baseline samples from 11 patients with a history of chemotherapy at other institute and four patients who had already undergone baseline sampling prior to treatment and for whom recurrence was confirmed during follow-up. Among the four patients who already underwent pretreatment sampling, ctDNA mutation was detected in only one. In this patient, the mutation spectrum was different between the pretreatment (*DDX3X* mutation detected) and relapsed (no mutation detected) samples.

*TP53*, *DDX3X*, and *BCOR* presented the highest mutation frequencies in the newly diagnosed patient group. Of note, patients with advanced-stage disease had more *DDX3X* and *BCOR* mutations (*P* = 0.01 and < 0.001, respectively) than early-stage and relapsed patient groups; thus, at least one genetic variation in the *TP53*, *DDX3X*, or *BCOR* was identified in all patients with advanced-stage disease (**Figure 2A**).

In 15 patients with relapse, eight (53.4%) harbored Tier I/II mutations (**Figure 2B**). The detection rate was lower than that for newly diagnosed patients, which might be partly related to the poor quality of samples or low DNA amounts obtained from relapsed patients who were heavily pre-treated prior to sampling. Nevertheless, *TP53* (33.0%) was the most commonly mutated gene, followed by *POT1* (13.0%).

### Feasibility of ctDNA monitoring in combination with EBV quantification

**Figure 3:**
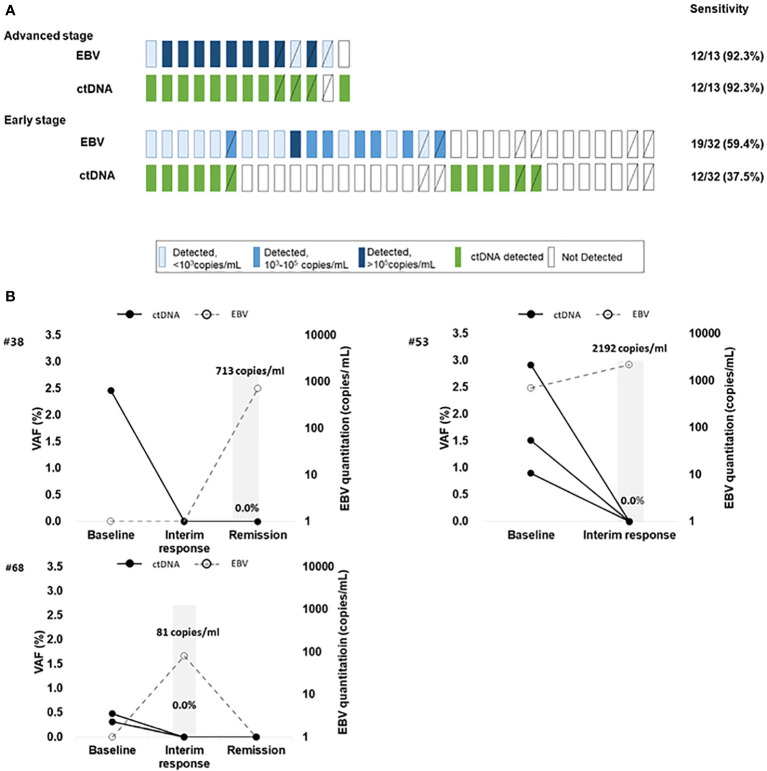
**(A)** The detection of ctDNA mutations and EBV DNA quantitation in ENKTL patients at baseline. The difference in detection of ctDNA mutation and EBV DNA according to disease stage. Diagonal lined boxes indicate patients with baseline sampling at the time of relapse **(B)** Three ENKTL patients with discrepant results between trackable ctDNA mutations and EBV DNA quantitation results who achieved complete disease remission Patient #53 had elevated plasma EBV DNA after concurrent chemo radiotherapy (CCRT). The patient showed a complete response to the therapy but died of sepsis; EBV DNA elevation in this patient could be attributed to causes other than ENKTL progression. Two patients (Patient #68 and patient #38) showed transient elevation of EBV during chemotherapy and there was no sign of residual disease judged by CT or patients’ clinical status, and the patients maintained remission without relapse. The bold black line indicates the mutation frequency changes in patients, and the dotted line indicates the EBV DNA quantitation results.

The 45 patients were classified as having advanced- or early-stage disease; the detection rates of baseline ctDNA mutations and EBV quantitation results in each patient were compared (**Figure 3A**). As expected, 84.6% (11/13) of patients in advanced stage were detected by both methods while EBV- and ctDNA mutation-positive rates in the early stage were low (19/32 and 12/32, respectively) with a low concordance. Whole blood EBV DNA was not detected in the baseline samples from 14 of 45 patients (31.3%), although, seven of 14 patients (one in stage IV and six in stage I/II) harbored ctDNA mutations that reflected their clinical course. This suggests that ctDNA genotyping can be a complementary method to EBV DNA quantification for disease monitoring, especially in EBV-negative patients. Among the 14 patients with EBV-positive and ctDNA mutation -negative results, 13 patients were at early stage, and the median EBV quantitation was 1021.5 copies/mL, which was approximately 1/10 of that for ctDNA-positive cases (median EBV quantitation, 14201 copies/mL).

In seven patients who had ctDNA mutation -positive results at baseline and achieved remission, none had mutations in plasma within 2–4 cycles of chemotherapy whereas EBV DNA was detected from three during monitoring (**Figure 3B**). This may suggest that EBV quantitation does not fully reflect patient’s disease status and ctDNA genotyping can be more specific.

**Figure 4:**
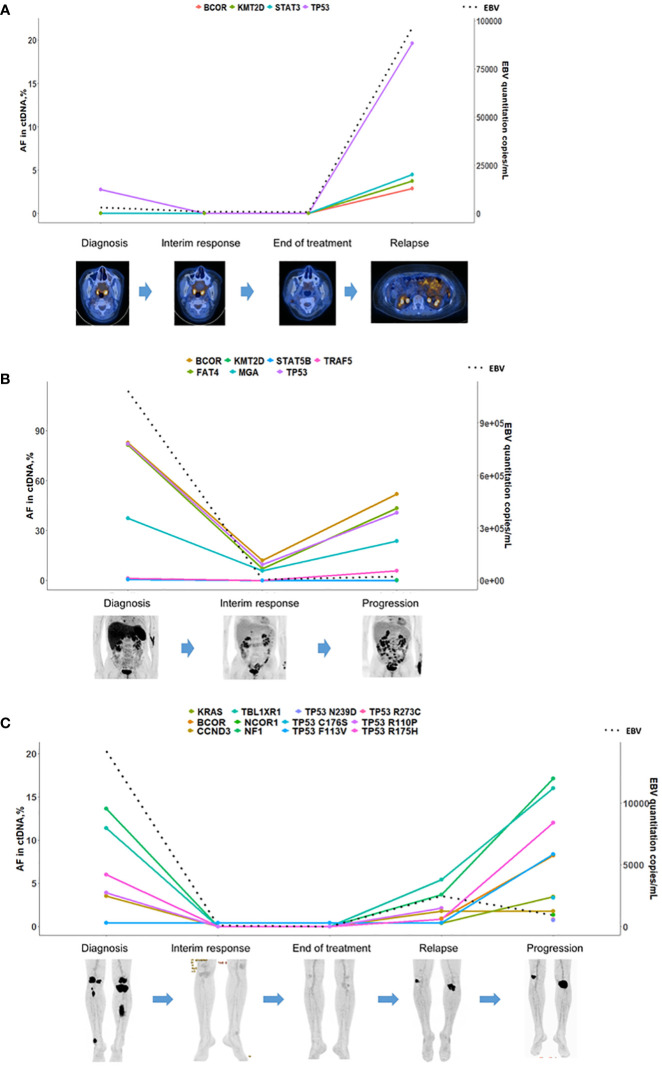
ctDNA mutation surveillance for disease progression/relapse in ENKTL patients. **(A)** A patient at stage 2 and in disease progression. The ctDNA mutation cleared before the complete remission assessed by PET/CT. The frequency of the *TP53* mutation, which was detected in the baseline sample, increased with a dramatic spike along with the other three emerging mutations at disease progression. **(B)** A relapsed patient treated with Pembrolizumab. The mutation burden correlated with the clinical course of the patient and decreased in a sample at partial response and increased in a sample at disease progression. **(C)** A patient at stage 4 who achieved complete remission was monitored thereafter. Plasma ctDNA mutation was detected in a sample at the third visit, and molecular clonal expansion was observed samples at the fourth visit and disease progression. The dotted line in the figures indicated the quantitative results of EBV-DNA.

Longitudinal plasma samples (n = 112) were serially collected from 39 patients in the cohort; Tier I/II mutations were identified in pre-treatment or relapsed/refractory samples (Additional file: **Table S5**) among 21 patients. A total of 14 patients experienced disease progression or relapse, and the majority (10/14, 71.4%) showed persistently detectable or emerging ctDNA mutations. An exemplary case with disease involving the liver and spleen showed an increase in VAFs of mutations as well as an increase of whole blood EBV DNA titer as the disease progressed (Additional file: **Figure S1A**). On the other hand, a case with localized disease involving the nasal cavity showed an absence of mutations and a low level of detectable EBV DNA; the VAFs of newly emerging mutations and EBV DNA titer were elevated as new lesions in the spleen appeared (Additional file: **Figure S1B**). Another exemplary case involving the nasopharynx showed a low VAF at diagnosis and the resolution of mutations after treatment. However, the patient had new intra-abdominal lesions without any lesions in the nasopharynx, with an increase of EBV DNA titer, re-appearance of initial mutations and emergence of new mutations (**Figure 4A**). Many cases showed frequent changes in the composition of mutations with each genetic mutation indicating the occurrence of clonal evolution (**Figures 4B, C**).

### Prognostic impact of ctDNA mutations

**Figure 5:**
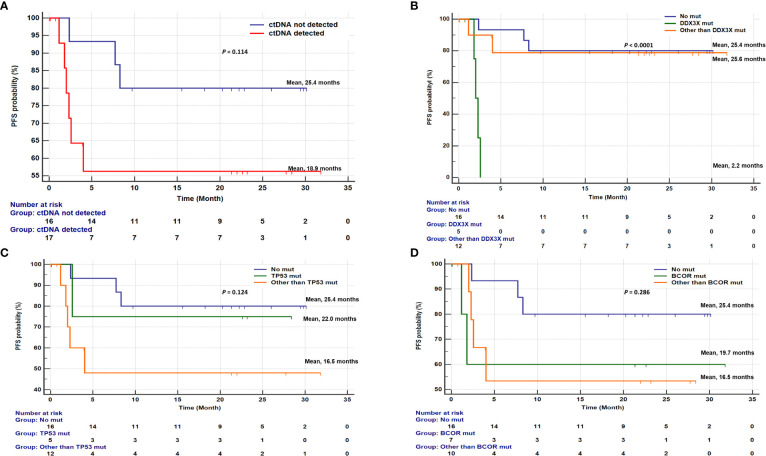
Kaplan-Meier curves for progression-free survival (PFS) of 33 newly diagnosed ENKTL patients. **(A)** Detection of ctDNA mutations in pretreatment blood sample was associated with progression-free survival (PFS) (median PFS, 18.9 months vs 25.4 months; P=0.114). Association of PFS between DDX3X, TP53, and BCOR gene mutations. **(B)** Comparison of PFS among patients without any mutation, with DDX3X mutation, and with mutations in other than DDX3X (median PFS, 25.4 months vs. 2.2 months vs. 25.6 months; P<0.0001). **(C)** Comparison of PFS among patients without any mutation, with TP53 mutation, and with mutations in other than TP53 (median PFS, 25.4 months vs. 22.0 months vs. 16.5 months; P=0.124). **(D)** Comparison of PFS among patients without any mutation, with BCOR mutation, and with mutations in other than BCOR (median PFS, 25.4 months vs. 19.7 months vs. 16.5 months; P=0.286).

Of the 34 newly diagnosed patients, one was excluded due to follow-up loss immediately after diagnosis, and the remaining 33 patients had a median follow-up period of 21.4 months (IQR; 4.7–25.1 months). Survival analysis showed that *DDX3X* and *BCOR* mutations tended to be associated with inferior OS, although this was not statistically significant (Additional file: **Figure S2**). By contrast, the median PFS of patients with *DDX3X* mutations (2.2 months) was shorter than that for patients with other gene mutations (25.6 months) or no mutation (25.4 months) (log-rank test; *P* < 0.0001, **Figure 5**).

Multivariable Cox regression analysis demonstrated an independent prognostic value of *DDX3X* mutation for PFS (hazard ratio [HR], 8.256; 95% confidence interval [95%CI], 1.371- 49.722; *P* = 0.021, **Table 2**).

**Table 2:** Analysis of prognostic factors for ENKTL patients.

	PFS				OS			
	Univariate	Multivariate*			Univariate	Multivariate*		
	*P*	OR	95%CI	*P*	*P*	OR	95%CI	*P*
Age (years)	0.618	–	–	–	0.272	–	–	–
*BCOR* mutation	0.477	–	–	–	0.144	–	–	0.288
*DDX3X* mutation	<0.001	8.256	1.371 to 49.722	0.021	0.228	–	–	–
*TP53* mutation	0.742	–	–	–	0.746	–	–	–
EBV DNA quantitation (copies/mL)	0.201	–	–	–	0.042	–	–	0.162
PINKE: 1 vs ≥2	0.006	11.79	1.309 to 106.147	0.028	0.043	14.837	1.861 to 118.28	0.011

*The variables that entered the multivariate Cox regression model included variables identified by univariate analysis with P <0.15.

PFS, progression-free survival; HR, hazard ratio; CI, confidence interval; PINK, prognostic index of natural killer lymphoma; PINK-E, prognostic index for natural killer cell lymphoma–Epstein-Barr virus; OS, overall survival.

## Discussion

The aggressiveness of ENKTL could differ according to the involved sites as well as the EBV DNA titer in blood; patients with tumors in non-nasal areas and high level of EBV DNA at diagnosis frequently show an aggressive clinical course, resulting in poor outcomes even in cases of localized disease. Therefore, an assessment of pre-treatment risk may be fundamental and influence the outcome of primary treatments, especially when most patients show unfavorable outcomes if the first-line treatment fails. Fluorodeoxyglucose (FDG)-PET/CT has been evaluated as a tool for risk prediction in lymphoma; however, its value is limited because ENKTL tumor cells are generally mixed with inflammatory cells, and combined inflammation may increase FDG uptake (21, 22). Furthermore, coagulative necrosis frequently found in ENKTL may also decrease FDG uptake. By contrast, EBV-DNA level in blood reflects the tumor burden and can be used for disease monitoring (6, 23, 24). However, EBV infection is highly prevalent in healthy individuals (25), and EBV DNA is frequently detected in elderly people (26). Therefore, EBV DNA level in blood may not be accurate for estimation of tumor burden in ENKTL patients. Although a previous study suggested that the Deauville score, reflecting FDG uptake and EBV DNA in blood, at the end of treatment could predict risk of relapse in ENKTL, its value is not sufficient to estimate treatment strategies for ENKTL (27).

In this study, we evaluated the feasibility and performance of in-depth cell-free ctDNA sequencing as a tool for prognostication and monitoring of ENKTL. We used a sensitive customized ctDNA NGS panel covering 171 genes, allowing detection of mutations with very low VAF (0.25%). The number of genes in the panel was larger and the detection sensitivity was higher than those in previous studies performed in NK or T-cell lymphomas (28, 29). The high-sensitivity of our platform allowed the detection of mutations repeatedly reported as main genetic alterations in ENKTL such as those in *TP53, KRAS, EP300*, and *MGA*, even those existing in very low VAFs.

Previous studies monitoring ENKTL patients have reported that clonal changes were as frequent as in other types of lymphoma (29, 30), suggesting that genomic profiling using proper multi-gene panels is needed both for initial diagnosis as well as monitoring. In this study, the three most frequently mutated genes (*BCOR*, *TP53*, and *DDX3X*) were found in all patients with advanced disease, in agreement with previous studies that have reported that mutations in *DDX3X* and *TP53* were adverse prognostic markers in ENKTL (10). *DDX3X*, a RNA helicase family gene, and *TP53*, a tumor suppressor gene, are assumed to have a close relationship with the biological process of ENKTL (8). Loss-of-function mutations of *DDX3X* are also frequent in B cell lymphomas including Burkitt lymphoma and DLBCL (31). *DDX3X* mutants exhibited loss of suppressive effects on cell-cycle progression in NK cells and transcriptional activation of pro-proliferative pathways in ENKTL (8, 32). Mutations of *DDX3X* is an adverse prognostic marker in CHOP-treated ENKTL patients, but this is not the case in ENKTL patients treated with asparaginase (ASP)-based regimens (10). In our study, all patients were treated with ASP-based regimens, yet the *DDX3X* mutation showed adverse effect on PFS in this study.

The *TP53* mutation was more frequent in relapsed patients (33.0%) than in newly diagnosed patients (18.0%). Considering that clonal evolution is frequent in lymphoma, most *TP53* mutations detected in relapsed/refractory patients may be from emerging clones during treatment that contribute to tumor relapse, as reported by previous studies in other lymphomas (33, 34). However, further studies including a larger number of serial samples from patients with ENKTL are needed to verify the clonal evolution of *TP53*.

**Figure 6:**
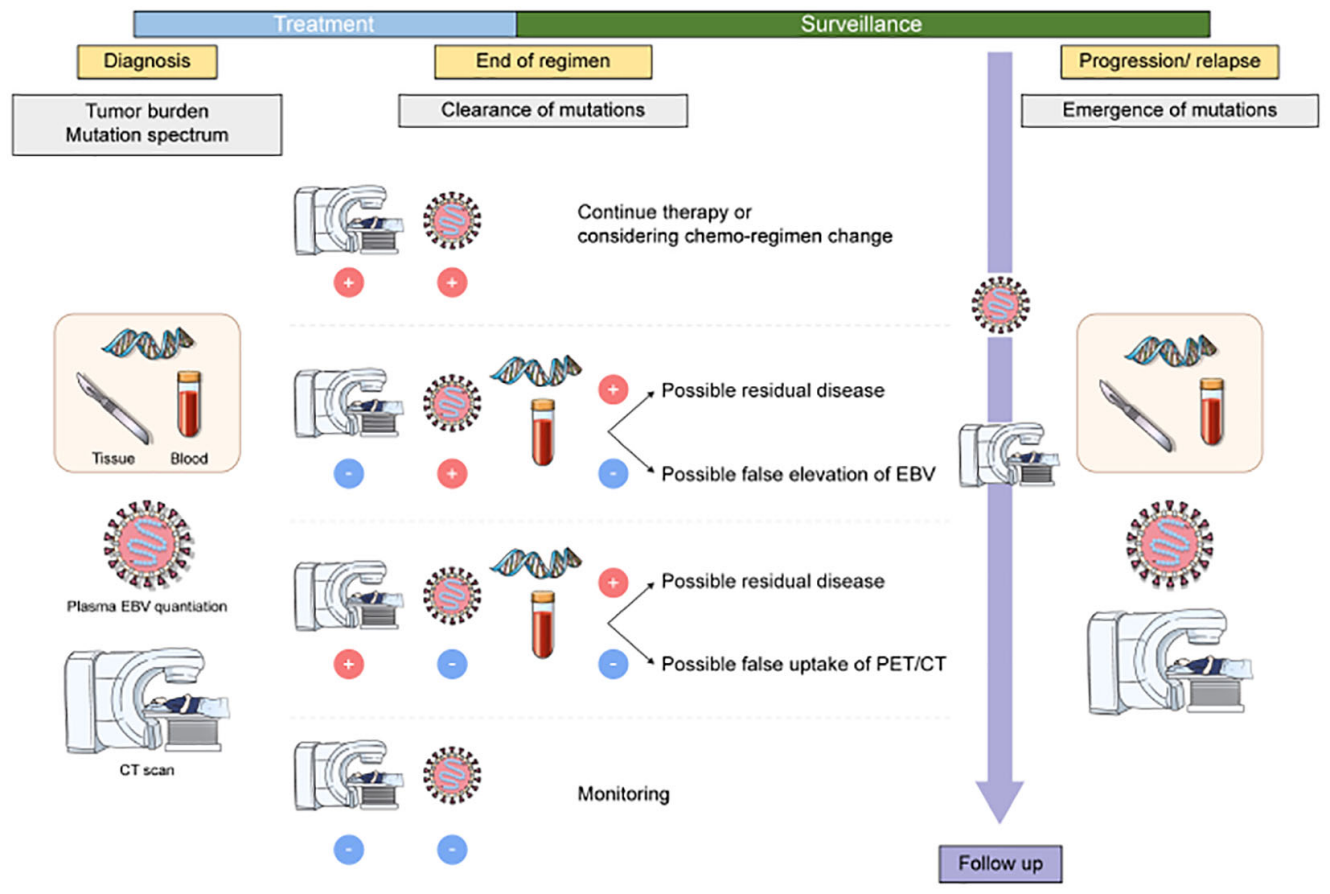
Application of liquid biopsy in patients with ENKTL. Upon diagnosis, baseline samples are collected. Once minimal residual disease (MRD) has been detected *via* liquid biopsy, further treatment options are considered. An additional series of samples can be collected during relapse to monitor changes in the patient, followed by initial treatment.

Herein, we demonstrated that ctDNA genotyping is a useful tool to diagnose ENKTL, estimate tumor burden, monitor treatment response, and detect emerging mutations of residual disease or relapse, and therefore, the assay may be suggested as an option for patient care in combination with conventional tools (**Figure 6**). Current diagnostic tools for ENKTL include imaging studies and EBV DNA level in blood. However, even if the treatment was very effective in imaging studies, uptake alterations sometimes remained and EBV DNA level could increase without clinical evidence of residuals or progression of ENKTL (26, 35, 36). Therefore, when the clinical features and results of the conventional monitoring tools conflict, a ctDNA analysis may help to estimate the patient’s condition and make therapeutic decision. Specifically, we found that the ctDNA genotyping could be used for tumor burden monitoring in EBV-negative patients. After chemotherapy or concurrent chemoradiotherapy (CCRT) treatments, ctDNA analysis may be performed along with imaging and EBV DNA quantitation. In patients showing discrepancies between imaging and EBV results, ctDNA genotyping may provide additional information for medical decisions. In such cases, if mutation is not detected in plasma sample (negative ctDNA), surveillance monitoring without additional chemotherapy and other causes of elevated EBV could be considered. However, if mutation is detected in plasma sample (positive ctDNA), a short-term follow-up or additional secondary therapy could be planned due to the possibility of residual ENKTL. By contrast, in patients with relapse, ctDNA analysis may indicate the necessity for a change in treatment regimen and allow more accurate prognoses based on changes in the mutation spectrum.

There were some limitations in our study. First, some of the tissue samples were analyzed by panels that targeted different genes; therefore, only genes targeted by both panels could be compared. Second, EBV DNA quantitation measurement was done in the whole blood samples. Studies reported that the plasma is a preferable sample than whole blood in measuring EBV DNA as a marker of EBV diseases (6, 37, 38). However, comprehensive recommendations and international guidelines are still lacking (39), and the institute where this study was conducted used whole blood samples for EBV quantitation in clinical practice that we used the whole blood sample. Thus, tumor burden analysis of ctDNA mutation and plasma EBV DNA quantitation might be needed in the future study. Third, although this study enrolled a larger number of patients with longitudinal samples than previous studies (16, 29), the sample size may still be too small to establish a firm conclusion. The topic should be further explored through a larger, prospective clinical trial. Nevertheless, ctDNA mutations showed dynamic properties during treatment, suggesting that they could be excellent indicators of disease course.

In conclusion, ctDNA testing at diagnosis may confirm the presence of a mutation that can be subsequently traced in blood samples during monitoring in patients with ENKTL. Testing it could act as an excellent marker providing additional information compared to that by imaging and EBV DNA quantitation and contribute to making effective medical decisions.

## Data availability statement

The original contributions presented in the study are included in the article/**Supplementary Material**. Further inquiries can be directed to the author (jjkim0810@yuhs.ac).

## Ethics statement

The studies involving human participants were reviewed and approved by Ethics Committee of Samsung Medical Center. The patients/participants provided their written informed consent to participate in this study.

## Author contributions

JK and H-YK designed the study, interpreted data, and wrote the manuscript; ZC, SH, and HJ performed the molecular studies; WK, SK, H-YK interpreted data; SY, collected data; SS designed the study and contributed to manuscript revision; SK, JC, and S-TL provided study materials and contributed to manuscript revision.
