# Supplementary material for: In-depth circulating tumor DNA sequencing for prognostication and monitoring in natural killer/T-cell lymphomas
Source: Front Oncol. 2023 Feb 10;13:1109715. doi: 10.3389/fonc.2023.1109715 (PMC9954142; doi:10.3389/fonc.2023.1109715)
Supplement: Supplementary file 1 [file DataSheet_1.pdf]

Supplementary materials

Supplementary Tables

Gene lists					
ALK	*CD274*	*EP300*	*KIT*	*PDE7B*	*SYK*
APC	*CD28*	*EPHA7*	*KLF2*	*PIK3CD*	*TBL1XR1*
ARID1A	*CD38*	*ETV6*	*KLHL14*	*PIK3R1*	*TCF3*
ARID1B	*CD58*	*EZH2*	*KMT2A*	*PIM1*	*TET1*
ARID2	*CD70*	*FAS*	*KMT2C*	*PLCG1*	*TET2*
ASXL3	*CD79A*	*FAT4*	*KMT2D*	*POT1*	*TNFAIP3*
ATM	*CD79B*	*FOXO1*	*KRAS*	*PRDM1*	*TNFRSF14*
ATP6AP1	*CDK4*	*FOXP1*	*M6PR*	*PRKCB*	*TNFRSF1B*
ATP6V1B2	*CDK6*	*FYN*	*MALT1*	*PRKCQ*	*TP53*
ATXN1	*CDKN1B*	*GATA3*	*MEF2B*	*PTEN*	*TP73*
ATXN7L1	*CDKN2A*	*GNA13*	*MGA*	*PTPN1*	*TRAF2*
B2M	*CDKN2B*	*GPR183*	*MGAM*	*PTPRC*	*TRAF3*
BAK1	*CDKN2C*	*HLA-A*	*MIR17HG*	*RB1*	*TRAF5*
BCL10	*CEBPA*	*HLA-B*	*MSH3*	*REL*	*UBR5*
BCL11A	*CHD8*	*HLA-C*	*MSN*	*RELA*	*VAV1*
BCL11B	*CHPF2*	*HNRNPA2B1*	*MYC*	*RHOA*	*WDR66*
BCL2	*CIITA*	*ID3*	*MYD88*	*RHOH*	*YTHDF2*
BCL6	*CMPK1*	*IDH2*	*NCOR1*	*RRAGC*	*ZAP70*
BCOR	*CREBBP*	*IKBKB*	*NF1*	*S1PR1*	*ZEB1*
BCORL1	*CSNK1A1*	*IKZF2*	*NFKB1*	*SETD1B*	*ZFP36L2*
BIRC3	*CSNK2A1*	*IL2RG*	*NFKB2*	*SETD2*	*ZNF638*
BRAF	*CSNK2B*	*IL6R*	*NFKBIA*	*SGK1*	
BTG1	*CTCF*	*IRF2BP2*	*NFKBIE*	*SKI*	
BTG2	*CTNNB1*	*IRF4*	*NOTCH1*	*SMARCA4*	
CARD11	*CXCR4*	*IRF8*	*NOTCH2*	*SOCS1*	
CBLB	*DDX3X*	*ITPKB*	*NRAS*	*SPEN*	
CCND1	*DNMT3A*	*JAK1*	*OSBPL10*	*STAT1*	
CCND3	*DTX1*	*JAK2*	*PAX5*	*STAT3*	
CCR4	*EBF1*	*JAK3*	*PDCD1*	*STAT5B*	
CCR7	*ECSIT*	*KDM6A*	*PDCD1LG2*	*STAT6*	

Supplementary Table S1. List of genes included in the ENTKL ctDNA panel.

Sample ID	Tissue						ctDNA					
	**ASCO/AMP Classification**	**Gene**	**Accession**	**exon**	**AA change**	**%VAF**	**ASCO/AMP Classification**	**Gene**	**Accession**	**exon**	**AA change**	**%VAF**
**50**	Tier 1/2	*BCOR*	NM_001123385.1	exon12	L1543X	93.3	Tier 1/2	*BCOR*	NM_001123385.1	exon12	L1543X	0.9
	Tier 1/2	*TBL1XR1*	NM_024665	exon4	p.S37F	43.7	Tier 1/2	*TBL1XR1*	NM_024665	exon4	p.S37F	11.4
	Tier 1/2	*TP53*	NM_000546.5	exon4	F113V	89.4	Tier 1/2	*TP53*	NM_000546.5	exon4	F113V	0.4
	Tier 1/2	*KRAS*	NM_033360.2	exon2	G12D	44.4	Tier 1/2	*KRAS*	NM_033360.2	exon2	G12D	0.4
	Tier 1/2	*NF1*	NM_000267	exon56	H2769D	44.3	Tier 1/2	*NF1*	NM_000267	exon56	H2769D	13.6
							Tier 1/2	*TP53*	NM_000546.5	exon5	R175H	0.8
							Tier 1/2	*TP53*	NM_000546.5	exon4	R110P	2.1
**61**	ND						ND					
**63**	Tier 1/2	*BCOR*	NM_001123385.1	exon13	W1598X	22.0	Tier 1/2	*BCOR*	NM_001123385.1	exon13	W1598X	2.2
	Tier 1/2	*BCOR*	NM_001123385.1	intron 12	c.4493+1G>A	10.6						
	Tier 1/2	*PTPRC*	NM_002838.5	intron 15	c.1721-1G>A	17.3						
	Tier 1/2	*PTPRC*	NM_002838.5	exon5	P140fs	15.3						
**65**	Tier 1/2	*DDX3X*	NM_001356.3	exon14	V513I	61.0	Tier 1/2	*DDX3X*	NM_001356.3	exon14	V513I	4.2
							Tier 1/2	*KRAS*	NM_033360	exon4	K117N	0.7
**67**	ND						ND					
**68**	ND						Tier 1/2	*EP300*	NM_001429	exon8	V550fs	0.3
							Tier 1/2	*MGA*	NM_001164273	exon18	C2339fs	0.5
**75**	ND						ND					
**37**	Tier 1/2	*MSN*	NM_002444.2	exon6	L192fs	14.6	ND					
	Tier 1/2	*KIT*	NM_000222.2	exon9	A507P	15.1						
**38**	ND						Tier 1/2	*TP53*	NM_000546.5	exon7	S241Y	2.5
**40**	ND						ND					
**42**	Tier 1/2	*TP53*	NM_000546.5	exon5	C176F	20.6	ND					
	Tier 1/2	*STAT3*	NM_139276.2	exon21	E638Q	24.3						

Supplementary Table S2. Somatic mutations detected in paired tissue and plasma samples from 11 patients.

Abbreviation: ND; not detected, AA; amino acid, VAF; variant allele frequency, ASCO/AMP; American Society of Clinical Oncology/American Molecular Pathology.

Sample type		Average depth	On target	% region covered (>100 unique molecular barcodes)	% region covered (>200 unique molecular barcodes)	% region covered (>300 unique molecular barcodes)	% region covered (>500 unique molecular barcodes)	% region covered (>1000 unique molecular barcodes)
Panel validation blood sample (N = 34)	Average	21937.7×	68.63%	99.06%	92.95%	83.48%	6.53%	0.35%
	SD	7557.6×	2.45%	0.24%	17.57%	27.84%	0.42%	0.39%
Cohort blood sample (N = 118)	Average	25076.9×	69.17%	99.04%	92.78%	83.39%	65.75%	47.63%
	SD	6043.7×	1.62%	2.47%	16.93%	28.77%	42.52%	43.82%
Tumor tissue (N = 4)	Average	3085.5×	66.55%	99.73%	94.25%	82.25%	67.70%	26.45%
	SD	876.2×	3.68%	0.48%	9.73%	28.30%	33.87%	17.71%

Supplemental Table S3. Sequencing statistics.

Cohort No.	Gene	NM	HGVSc	HGVSp	VAF	Detection with driver mutations	Disease progression
31	*DNMT3A*	NM_022552.4	c.1903C>T	p.Arg635Trp	1.0%	No	No
34	*JAK3*	NM_000215.3	c.1533G>A	p.Met511Ile	0.4%	No	No
37	*STAT5B*	NM_012448.3	c.598C>T	p.Arg200Trp	2.2%	No	No
40	*ATM*	NM_000051.3	c.5051C>T	p.Ser1684Phe	0.3%	No	No
	CCR7	NM_001838.3	c.917T>C	p.Ile306Thr	0.8%		
	DNMT3A	NM_022552.4	c.2054G>T	p.Gly685Val	0.5%		
43	*DNMT3A*	NM_022552.4	c.1319G>A	p.Trp440Ter	0.5%	Yes	Yes
46	*KMT2C*	NM_170606.2	c.1181G>A	p.Cys394Tyr	5.4%	Yes	No
48	*DNMT3A*	NM_022552.4	c.2257T>C	p.Trp753Arg	3.3%	Yes	Yes
50	*ARID1B*	NM_020732.3	c.986C>G	p.Ala329Gly	7.2%	Yes	Yes
51	*DNMT3A*	NM_022552.4	c.1668-1G>T		4.5%	Yes	Yes
	DNMT3A	NM_022552.4	c.2386G>C	p.Gly796Arg	2.0%		
53	*DNMT3A*	NM_022552.4	c.2204A>G	p.Tyr735Cys	0.3%	Yes	Unknown
55	*KMT2C*	NM_170606.2	c.2618A>T	p.Gln873Leu	3.5%	No	No
	DNMT3A	NM_022552.4	c.905G>A	p.Gly302Asp	0.1%		
56	*DNMT3A*	NM_022552.4	c.2311C>T	p.Arg771Ter	0.9%	No	No
61	*DNMT3A*	NM_022552.4	c.1916T>C	p.Leu639Pro	0.6%	No	No
62	*DNMT3A*	NM_022552.4	c.2210T>G	p.Leu737Arg	1.8%	Yes	No
63	*KMT2C*	NM_170606.2	c.1154G>T	p.Cys385Phe	5.9%	Yes	Yes
70	*DNMT3A*	NM_022552.4	c.2073dup	p.Gln692ThrfsTer21	0.7%	Yes	Yes
	DNMT3A	NM_022552.4	c.2309C>T	p.Ser770Leu	0.5%		

Supplementary Table S4. Detected clonal hematopoietic indeterminate potential (CHIP) variants in 34 newly diagnosed ENKTL patients.

Abbreviation: VAF; variant allele frequency,

Patient number	Time point	Gene	NM	exon	aa Change	Variant frequency (%)	EBV quantitation (copies/mL)
**31**	A						589
**31**	B						251
**31**	C						0
**33**		*ETV6*	NM_001987	exon1	p.Q7fs	29.52	
**33**		*CARD11*	NM_032415	exon5	p.G123S	31.80	
**33**	A	*PIM1*	NM_002648.3	exon4	p.V86M	2.52	577
**33**		*CREBBP*	NM_004380.2	exon26	p.R1446C	63.05	
**33**		*MYD88*	NM_002468.4	exon5	L265P	44.09	
**33**	D	*NRAS*	NM_002524	exon3	p.Q61R	0.25	3373
**34**		*BRAF*	NM_004333	exon15	p.K601N	2.39	
**34**		*JAK3*	NM_000215	exon13	p.A573V	15.03	
**34**	A	*BCOR*	NM_001123385.1	exon4	p.T817fs	47.79	0
**34**		*KMT2D*	NM_003482	intron 28	c.6110-1G>A	25.79	
**34**	B						0
**34**	C						0
**35**	A	*ARID2*	NM_152641	exon19	p.M1742fs	3.19	0
**35**	B						0
**35**	C						0
**36**	A						74
**36**	D						14719
**37**	A						0
**37**	B						0
**37**	C						0
**38**	A	*TP53*	NM_000546.5	exon7	p.S241Y	2.47	0
**38**	B						81
**38**	C						0
**39**	A						422
**39**	D	*NRAS*	NM_002524	exon2	p.G13D	5.99	77125
**39**		*NRAS*	NM_002524	exon2	p.G13C	4.10	

**39**		*POT1*	NM_015450	exon8	p.G108X	19.27	
**39**		*KRAS*	NM_033360	exon3	p.Q61H	0.50	
**39**		*KRAS*	NM_033360	exon3	p.A59E	0.67	
**39**		*KRAS*	NM_033360	exon2	p.G12S	29.57	
**40**	A						133
**40**	B						251
**40**	C						96
**41**	A						466259
**42**	A						0
**42**	B						0
**42**	C						0
**43**	A	*DDX3X*	NM_001356.3	exon11	p.Q390fs	5.06	688
**43**	D						1947
**43**	D2						1302
**44**	A	*NFKB1*	NM_003998.3	exon15	p.E504X	4.58	201599
**44**		*NF1*	NM_000267	exon21	p.A805fs	0.24	
**44**		*DDX3X*	NM_001356.3	exon14	p.T532M	3.56	
**44**		*KMT2D*	NM_003482	exon11	p.E1076fs	3.94	
**45**	A						0
**45**	B						0
**45**	C						0
**46**	A	*KRAS*	NM_033360	exon2	p.Q22K	0.48	0
**46**	B						0
**46**	C						0
**47**	A	*TP53*	NM_000546.5	exon7	p.S241Y	2.76	3041
**47**	B						874
**47**	C						806
**47**	D	*BCOR*	NM_001123385.1	exon8	p.R1217X	2.87	96478
**47**		*STAT3*	NM_003150	exon20	p.S614R	4.49	
**47**		*KMT2D*	NM_003482	intron 35	c.10356-1G>T	3.75	
**47**		*TP53*	NM_000546.5	exon7	p.S241Y	19.61	
**48**	A	*KMT2A*	NM_001197104	exon18	p.S1786X	0.41	0

**48**		*DNMT3A*	NM_022552	exon15	p.W564R	3.28	0
**48**	D	*DNMT3A*	NM_022552	exon15	p.W564R	1.46	71
**49**	A						431
**49**	B						180
**49**	C						856
**49**		*POT1*	NM_015450	exon12	p.Q210X	34.57	
**49**	D	*EP300*	NM_001429	exon17	p.R1055X	0.40	964
**49**		*TP53*	NM_000546.5	exon8	p.D281N	66.27	
**50**		*TBL1XR1*	NM_024665	exon4	p.S37F	11.37	
**50**		*NF1*	NM_000267	exon56	p.H2769D	13.65	
**50**	A	*CCND3*	NM_001760	exon4	p.P212S	3.52	14201
**50**		*TP53*	NM_000546.5	exon5	p.R175H	6.01	
**50**		*TP53*	NM_000546.5	exon4	p.R110P	3.89	
**50**	B						81
**50**	C						0
**50**		*TBL1XR1*	NM_024665	exon4	p.S37F	5.43	
**50**		*NF1*	NM_000267	exon56	p.H2769D	3.67	
**50**		*TP53*	NM_000546.5	exon5	p.R175H	0.83	
**50**	D	*TP53*	NM_000546.5	exon4	p.R110P	2.13	2461
**50**		*KRAS*	NM_033360	exon2	p.G12D	0.37	
**50**		*BCOR*	NM_001123385.1	exon12	p.L1543X	0.91	
**50**		*TP53*	NM_000546.5	exon4	p.F113V	0.44	
**50**		*NCOR1*	NM_006311	exon46	p.E2330G	1.33	
**50**		*TBL1XR1*	NM_024665	exon4	p.S37F	16.00	
**50**		*KRAS*	NM_033360	exon2	p.G12D	3.43	
**50**		*NF1*	NM_000267	exon56	p.H2769D	17.12	
**50**		*BCOR*	NM_001123385.1	exon12	p.L1543X	8.22	
**50**	D2	*CCND3*	NM_001760	exon4	p.P212S	1.76	924
**50**		*TP53*	NM_000546.5	exon8	p.R273C	0.80	
**50**		*TP53*	NM_000546.5	exon7	p.N239D	0.71	
**50**		*TP53*	NM_000546.5	exon5	p.C176S	3.34	
**50**		*TP53*	NM_000546.5	exon5	p.R175H	12.00	

**50**		*TP53*	NM_000546.5	exon4	p.F113V	8.35	
**51**	A	*TP53*	NM_000546.5	exon8	p.R282W	0.98	264
**51**	B						0
**51**	C						0
**52**	A						20900
**52**	B						40074
**52**	C						27875
**53**		*TET2*	NM_001127208	exon11	p.A1840fs	0.90	
**53**	A	*B2M*	NM_004048	exon2	p.W115fs	1.51	688
**53**		*MSN*	NM_002444	exon8	p.E301fs	2.92	
**53**	B						2192
**54**		*KMT2C*	NM_170606	exon14	p.K827Q	4.12	
**54**		*CDKN2A*	NM_000077	exon2	p.E88X	33.23	
**54**	A	*NF1*	NM_000267	exon36	p.S1733F	20.68	19834
**54**		*BCOR*	NM_001123385.1	exon4	p.A184fs	20.63	
**54**		*DDX3X*	NM_001356.3	exon13	p.F447V	22.15	
**54**		*KMT2D*	NM_003482	exon10	p.R466fs	20.18	
**54**		*KMT2C*	NM_170606	exon14	p.K827Q	6.60	
**54**		*CDKN2A*	NM_000077	exon2	p.E88X	45.13	
**54**	D	*NF1*	NM_000267	exon36	p.S1733F	31.25	71920
**54**		*BCOR*	NM_001123385.1	exon4	p.A184fs	24.06	
**54**		*DDX3X*	NM_001356.3	exon13	p.F447V	26.98	
**54**		*KMT2D*	NM_003482	exon10	p.R466fs	40.14	
**55**		*FAT4*	NM_001291285	exon9	p.V3184I	81.55	
**55**		*TRAF5*	NM_001033910.2	intron 9	c.931-5G>A	1.19	
**55**	A	*BCOR*	NM_001123385.1	exon12	p.S1551fs	82.65	1081249
**55**		*STAT5B*	NM_012448	exon16	p.N642H	0.55	
**55**		*MGA*	NM_001164273	intron 9	c.3430+5G>T	37.38	
**55**		*TP53*	NM_000546.5	exon4	p.W53X	81.99	
**55**		*TP53*	NM_000546.5	exon4	p.W53X	9.59	
**55**	B	*BCOR*	NM_001123385.1	exon12	p.S1551fs	12.03	5292
**55**		*MGA*	NM_001164273	intron 9	c.3430+5G>T	5.72	

**55**	D	*FAT4*	NM_001291285	exon9	p.V3184I	6.90	20166
**55**		*FAT4*	NM_001291285	exon9	p.V3184I	43.46	
**55**		*TRAF5*	NM_001033910.2	intron 9	c.931-5G>A	5.65	
**55**		*BCOR*	NM_001123385.1	exon12	p.S1551fs	51.93	
**55**		*MGA*	NM_001164273	intron 9	c.3430+5G>T	23.70	
**55**		*KMT2D*	NM_003482	exon37	p.R3491C	0.17	
**55**		*TP53*	NM_000546.5	exon4	p.W53X	40.66	
**56**	A	*TNFAIP3*	NM_001270507	exon9	p.R706X	0.36	299928
**56**		*KMT2C*	NM_170606	exon15	p.Q873L	5.72	
**56**		*BCOR*	NM_001123385.1	exon12	p.S1551fs	30.34	
**56**		*TP53*	NM_000546.5	exon4	p.W53X	18.62	
**57**	A	*TNFAIP3*	NM_001270507	exon7	p.Q415fs	0.89	422
**57**		*BCOR*	NM_001123385.1	exon4	p.E812X	3.16	
**57**		*STAT3*	NM_003150	exon20	p.S614R	1.41	
**57**		*TP53*	NM_000546.5	exon7	p.D259N	0.15	
**57**	B						0
**57**	C						0
**58**	A						2058
**58**	B						164
**58**	C						251
**59**	A	*STAT3*	NM_003150	exon21	p.D661V	1.07	0
**59**		*TP53*	NM_000546.5	exon7	p.G244D	1.22	
**59**	B						0
**59**	C						0
**60**	A						1996
**60**	B						1742
**60**	C						0
**61**	A						177
**61**	B						0
**61**	C						0
**62**	A						1215
**62**	B						0

**62**	C						0
**63**	A	*BCOR*	NM_001123385.1	exon13	p.W1598X	2.22	161
**63**	B						0
**63**	C						0
**64**	A						0
**64**	B						0
**64**	C						0
**65**	A	*KRAS*	NM_033360	exon4	p.K117N	0.74	115224
**65**		*DDX3X*	NM_001356.3	exon14	p.V513I	4.21	
**65**	D	*KRAS*	NM_033360	exon4	p.K117N	0.24	520338
**65**		*DDX3X*	NM_001356.3	exon14	p.V513I	8.65	
**66**	A	*BCOR*	NM_001123385.1	exon8	p.R1217X	88.99	667632
**66**		*KMT2D*	NM_003482	exon48	p.R5179H	45.17	
**67**	A						3922
**67**	C						3931
**67**	D						20330
**68**	A	*EP300*	NM_001429	exon8	p.V550fs	0.32	0
**68**		*MGA*	NM_001164273	exon18	p.C2339fs	0.48	
**68**	B						81
**68**	C						0
**68**	C2						0
**69**	A						828
**69**	C						0
**69**	D						3767
**70**	A	*KRAS*	NM_033360	exon4	p.A146V	20.08	99541
**70**		*BCOR*	NM_001123385.1	exon7	p.R1163X	39.60	
**70**		*STAT5B*	NM_012448	exon16	p.N642H	31.66	
**70**		*CD79B*	NM_021602	exon4	p.Y92S	0.41	
**70**		*MSN*	NM_002444	intron 3	c.192+1G>A	35.16	
**70**	B						20600
**71**	A						2483
**71**	B						5050

**71**	C						2040
**72**	A						0
**73**		*DDX3X*	NM_001356.3	intron 2	c.103+1G>A	71.81	
**73**	A	*PIK3R1*	NM_181523.2	intron 13	c.1746-1G>A	31.81	6464805
**73**		*TP53*	NM_000546.5	exon6	p.R213X	66.32	
**73**		*DDX3X*	NM_001356.3	intron 2	c.103+1G>A	6.75	
**73**	D	*TP53*	NM_000546.5	exon8	p.R282fs	3.29	1138
**73**		*TP53*	NM_000546.5	exon7	p.R248Q	0.66	
**74**	A						0
**74**	B						0
**75**	A						0
**75**	B						0
**75**	C						0
**76**	A	*KDM6A*	NM_001291418	exon15	p.Q674X	2.79	3236
**76**		*TP53*	NM_000546.5	exon7	p.C238Y	2.80	

Supplementary Table S5. Mutations detected in the consecutive blood samples of the 45 ENKTL patients

ctDNA mutations detected in monitoring time point among patients; A as baseline, B as interim during follow up, C as remission state, and D as relapsed state. Matched EBV DNA quantitation results are also presented.

Abbreviation: aa; amino acid

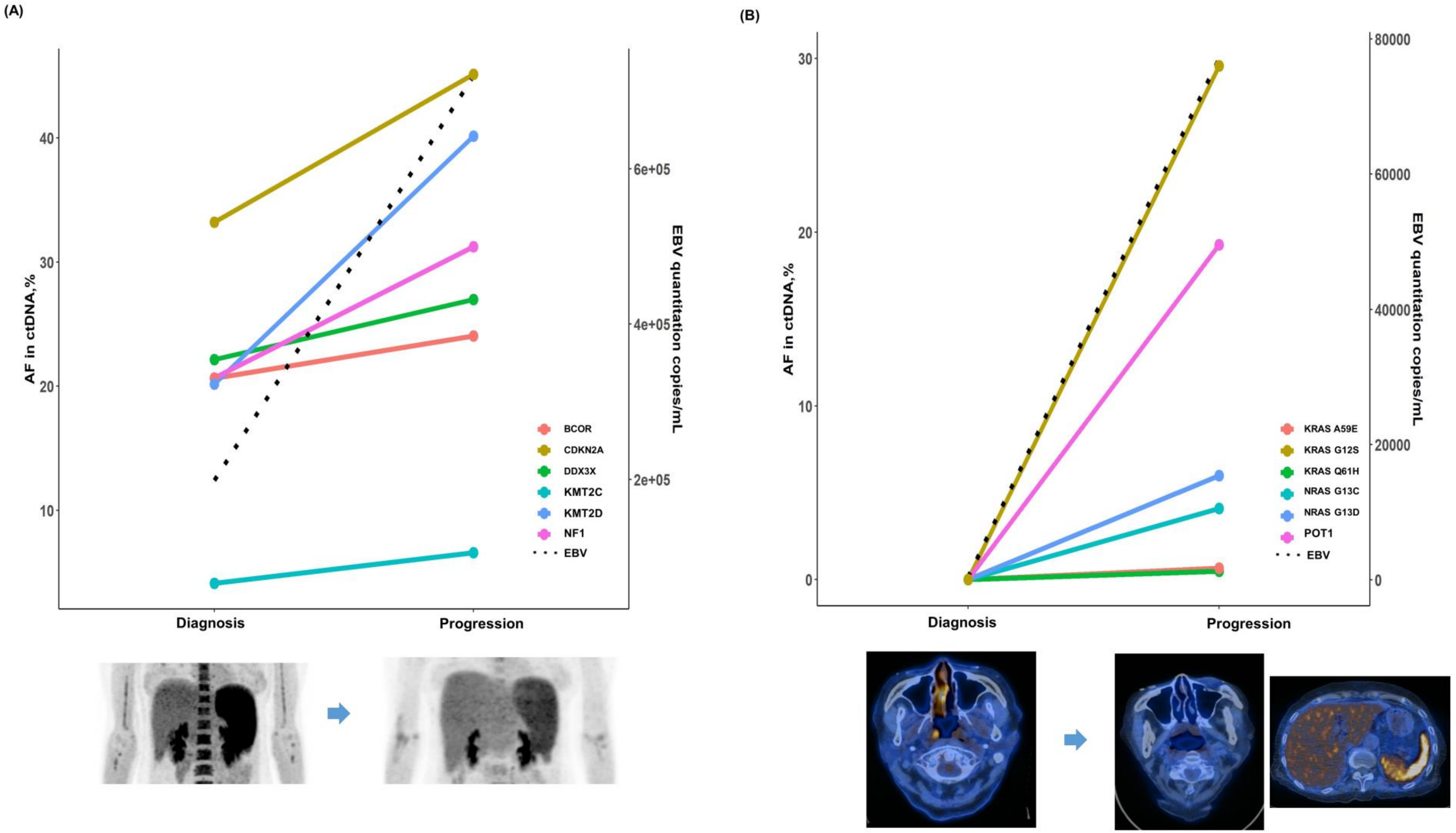
(A)
AF in ctDNA,%
EBV quantitation copies/mL
40
30
20
10
6e+05
4e+05
2e+05
BCOR
CDKN2A
DDX3X
KMT2C
KMT2D
NF1
EBV
Diagnosis
Progression
(B)
AF in ctDNA,%
EBV quantitation copies/mL
30
20
10
0
80000
60000
40000
20000
0
KRAS A59E
KRAS G12S
KRAS Q61H
NRAS G13C
NRAS G13D
POT1
EBV
Diagnosis
Progression

Supplementary Figure S1.

(A) Stage 4 ENKTL patient treated for concomitant haemophagocytic lymphohistiocytosis (HLH) until the first visit. The patient had progression based on PET/CT. Disease burden is expected to increase by ctDNA mutation variant frequency and EBV quantitation. **(B)** Stage 2 ENKTL patient with concurrent chemo-radiotherapy. The disease showed progression at the first visit. In this case, the baseline plasma sample had no ctDNA mutation, but emerged mutations were detected in the progressed sample.

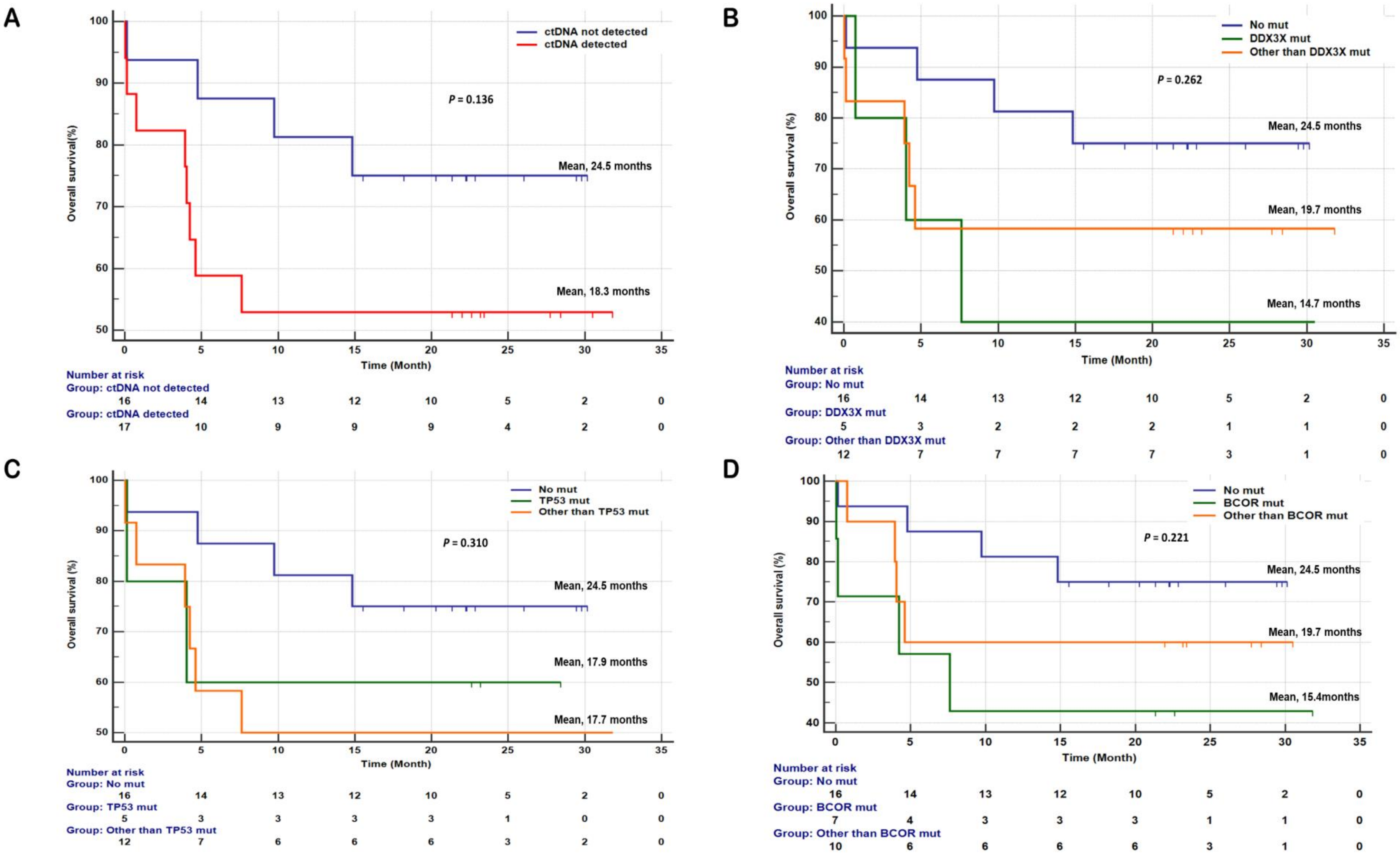

Supplementary Figure S2. Kaplan-Meier curves for overall survival (OS) of 33 newly diagnosed ENKTL patients.

(A) Detection of ctDNA mutations in pretreatment blood sample was associated with overall survival (OS) (median OS, 18.3 months vs. 24.5 months; *P*=0.136). (B)

Comparison of OS among patients without any mutation, with *DDX3X* gene mutation, and mutations other than *DDX3X* gene mutation group (median OS, 24.5 months vs. 14.7 months vs. 19.7 months; *P*=0.262). (C) Comparison of OS among patients without any mutation, with *TP53* gene mutation, and mutations other than *TP53* gene mutation group group (median OS, 24.5 months vs. 17.9 months vs. 17.7 months; *P*=0.310). (D) Comparison of OS among patients without any mutation, with *BCOR* gene mutation, and mutations other than *BCOR* gene mutation group (median OS, 24.5 months vs. 15.4 months vs. 19.7 months; *P*=0.221).
